# COVID-19-Associated Mucormycosis (CAM): Case-Series and Global Analysis of Mortality Risk Factors

**DOI:** 10.3390/jof7100837

**Published:** 2021-10-07

**Authors:** Abanoub Riad, Alshaimaa Ahmed Shabaan, Julien Issa, Sally Ibrahim, Hatem Amer, Yossef Mansy, Islam Kassem, Amira Bisher Kassem, Hans-Peter Howaldt, Miloslav Klugar, Sameh Attia

**Affiliations:** 1 Czech National Centre for Evidence-Based Healthcare and Knowledge Translation (Cochrane Czech Republic, Czech EBHC: JBI Centre of Excellence, Masaryk University GRADE Centre), Institute of Biostatistics and Analyses, Faculty of Medicine, Masaryk University, 625 00 Brno, Czech Republic; klugar@med.muni.cz; 2 Department of Public Health, Faculty of Medicine, Masaryk University, 625 00 Brno, Czech Republic; 3 Department of Oral and Maxillofacial Surgery, Faculty of Dentistry, Fayoum University, Fayoum 635 14, Egypt; aas16@fayoum.edu.eg; 4 Department of Biomaterials and Experimental Dentistry, Poznań University of Medical Sciences, 60-781 Poznan, Poland; julien.issa@student.ump.edu.pl; 5 Department of Oral and Maxillofacial Pathology, Faculty of Dentistry, Fayoum University, Fayoum 635 14, Egypt; sim11@fayoum.edu.eg; 6 Department of Oral and Maxillofacial Pathology, Faculty of Dentistry, Cairo University, Cairo 115 62, Egypt; hatemamer@dentistry.cu.edu.eg; 7 Department of Oral and Maxillofacial Surgery, Maadi Military Hospital, Cairo 117 11, Egypt; elmansi76@gmail.com; 8 Private Oral and Maxillofacial Surgery Practice, Alexandria 215 54, Egypt; ikassem@dr.com; 9 Department of Clinical Pharmacy and Pharmacy Practice, Faculty of Pharmacy, Damanhur University, Damanhur 225 11, Egypt; amira.kassem@pharm.dmu.edu.eg; 10 Department of Oral and Maxillofacial Surgery, Justus-Liebig-University, Klinikstrasse 33, 35392 Giessen, Germany; hp.howaldt@uniklinikum-giessen.de

**Keywords:** co-infection, COVID-19, cross-infection, Mucorales, mucormycosis, mycoses, review, *Rhizopus*, risk factors, steroids

## Abstract

Background: Since the novel coronavirus disease (COVID-19) outbreak, the cases of COVID-19 co-infections have been increasingly reported worldwide. Mucormycosis, an opportunistic fungal infection caused by members of the Mucorales order, had been frequently isolated in severely and critically ill COVID-19 patients. Methods: Initially, the anamnestic, clinical, and paraclinical features of seven COVID-19-associated mucormycosis (CAM) cases from Egypt were thoroughly reported. Subsequently, an extensive review of the literature was carried out to describe the characteristics of CAM cases globally, aiming to explore the potential risk factors of mortality in CAM patients. Results: Out of the seven reported patients in the case series, five (71.4%) were males, six (85.7%) had diabetes mellitus, and three (42.9%) had cardiovascular disease. All patients exhibited various forms of facial deformities under the computed tomography scanning, and two of them tested positive for Mucorales using the polymerase chain reaction (PCR) testing. Liposomal amphotericin B (LAmB) was prescribed to all cases, and none of them died until the end of the follow-up. On reviewing the literature, 191 cases were reported worldwide, of which 74.4% were males, 83.2% were from low-middle income countries, and 51.4% were aged 55 years old or below. Diabetes mellitus (79.1%), chronic hypertension (30%), and renal disease/failure (13.6%) were the most common medical comorbidities, while steroids (64.5%) were the most frequently prescribed medication for COVID-19, followed by *Remdesivir* (18.2%), antibiotics (12.7%), and *Tocilizumab* (5.5%). Conclusions: As the majority of the included studies were observational studies, the obtained evidence needs to be interpreted carefully. Diabetes, steroids, and Remdesivir were not associated with increased mortality risk, thus confirming that steroids used to manage severe and critical COVID-19 patients should not be discontinued. Lung involvement, bilateral manifestation, and *Rhizopus* isolation were associated with increased mortality risk, thus confirming that proactive screening is imperative, especially for critically ill patients. Finally, surgical management and antimycotic medications, e.g., amphotericin B and posaconazole, were associated with decreased mortality risk, thus confirming their effectiveness.

## 1. Introduction

Since the novel coronavirus disease (COVID-19) outbreak, the cases of COVID-19 co-infections have been increasingly reported worldwide from various clinical settings [1,2,3,4,5,6]. *Aspergillus* and *Candida* were the primary fungal genera responsible for mycosis in COVID-19 patients in severe and critical conditions, especially those with other medical risk factors [3,4,5,6,7,8]. Additionally, other less common fungal genera, e.g., *Cryptococcus* and *Mucor*, had been identified in combination with severe acute respiratory syndrome coronavirus-2 (SARS-CoV-2) infection [6,9].

Mucormycosis is an opportunistic fungal infection caused by species from the Mucorales order [10]. The commonly isolated species in the infected patients belong to three main genera; *Rhizopus*, *Lichtheimia* and *Mucor* [11,12]. The distribution of species responsible for mucormycosis differs geographically. The clinical forms of mucormycosis are also dependent on the causative species, e.g., members of the genera *Apophysomyces* and *Saksenaea* are believed to be responsible for cutaneous mucormycosis, and they are commonly found in Asian countries [13,14,15,16,17].

The clinical manifestation of mucormycosis invasion is variable because it progresses rapidly into the body system, and it can affect the paranasal sinuses, orbits, lungs, kidneys, central nervous system, gastrointestinal system, and skin [12,18,19,20]. In general, mucormycosis manifestations depend on two factors: (a) the route of entry of fungal spores into the human body, mainly by inhalation followed by ingestion or direct skin inoculation; (b) the predisposing disease of the infected patients [12]. Globally, diabetes mellitus is the most reported predisposing disease in mucormycosis patients in addition to other diseases, malignancies and organ transplantation [11]. Furthermore, corticosteroids and immunosuppressive therapy are considered as mucormycosis risk factors [21].

Generally, mucormycosis patients are treated by early identification and monitoring risk factors, followed by proper surgical intervention and administration of the appropriate antifungal medication. According to the European Society for Clinical Microbiology and Infectious Diseases (ESCMID) and the European Confederation of Medical Mycology (ECMM), liposomal amphotericin B (LAmB) is the most efficient antifungal drug for treating mucormycosis with a minimum dosage of 5 mg/kg/day [22,23]. Moreover, posaconazole and isavuconazole are recommended for refractory cases and those intolerant towards previous antifungals [23].

COVID-19 had been imposing significant challenges to both health systems and individuals, and one of these challenges is the lack of definitive clinical symptoms that can help in its diagnosis and triage. In addition to the common respiratory symptoms that could be confused with other respiratory diseases, COVID-19 patients reported a wide array of gastrointestinal, neurologic, dermatologic, and even oral manifestations, e.g., anosmia dysgeusia, ulcers, and mucositis [24,25,26,27,28,29,30,31,32,33,34,35,36,37]. The non-respiratory manifestations that occurred in COVID-19 patients were attributed to various pathophysiologic pathways, including the superinfection (or secondary infection) caused by opportunistic pathogens, e.g., moulds [38].

Lately, the incidence of mucormycosis in patients suffering or recovering from COVID-19 has been increasingly reported worldwide [9]. COVID-19 patient provides a suitable environment to promote mucormycosis. Most patients present a low oxygen level, hyperglycemic state, an acidic medium, high iron levels, and a decreased phagocytic activity. These conditions, associated with several other risk factors, promote opportunistic fungal infections such as mucormycosis [39].

Relying on the circumstances mentioned above, we report a series of seven COVID-19-associated mucormycosis (CAM) cases that have been diagnosed in Egypt. In addition, we aimed in this review to discuss our results in the context of existing evidence, so we performed an extensive review analysing the demographic, anamnestic and clinical characteristics, case management and outcomes of the CAM incidence and mortality.

## 2. Materials and Methods

### 2.1. Clinical and Paraclinical Examination

Between April and May 2021, seven cases of COVID-19 were referred to Gamal Abdel Nasser Hospital (Alexandria, Egypt), Maadi Military Hospital (Cairo, Egypt), Alexandria University Main Hospital (Alexandria, Egypt), and Fayoum University Hospital (Fayoum, Egypt) for clinical symptoms suggestive for mucormycosis infection. A thorough anamnestic investigation and a complete oral and maxillofacial clinical examination were initially conducted to justify the paraclinical examination. The radiographic assessment with cone-beam computed tomography (CBCT) and the microbiological and histopathological evaluations using biopsy specimens were utilised to reach a definite diagnosis in all the suspected cases.

### 2.2. Literature Review

An electronic search was executed using a combination of keywords (“ mucormycosis OR Mucorales OR black fungus” AND “COVID-19 OR SARS-CoV-2 OR coronavirus”) in Ovid MEDLINE^®^, Embase, and Google Scholar. Initially, the retrieved records were screened for their potential inclusion eligibility based on their titles and abstracts. Subsequently, the potentially eligible studies were thoroughly examined based on their full text to ensure their eligibility by having the *P* (population) *E* (exposure of interest) *O* (outcome) elements of aetiology and risk reviews. The *P* mnemonic referred to COVID-19 patients or survivors, the *E* mnemonic referred to the risk factors that may lead to mucormycosis infection, and the *O* mnemonic referred to the incidence of mucormycosis infection, which is diagnosed according to the European Organization for Research and Treatment of Cancer (EORTC) and ECMM [40,41,42]. No restrictions on language, publication date, or study type were applied.

### 2.3. Ethical Considerations

The case series part of the study was approved by the Fayoum University Supreme Committee for Scientific Research Ethics (FU-SCSRE) under the approval code EC 2131 on 11 April 2021. All the included cases gave their informed consent to share their clinical data and images for scientific purposes.

The clinical cases were followed and reported according to the CARE guidelines for case reports [43]. The whole study was carried out according to the Declaration of Helsinki for research involving human subjects [44].

### 2.4. Statistical Analysis

All statistical tests were executed by using the Statistical Package for the Social Sciences (SPSS) version 27.0 (SPSS Inc. Chicago, IL, USA, 2020) [45]. Primarily, descriptive statistics were performed for the demographic, medical, COVID-19-related, and mucormycosis-related data using frequencies (*n*), percentages (%), and central tendency measures such as mean (*μ*) and standard deviation (*SD*).

Consequently, inferential statistics using a chi-squared test (*χ^2^*), Fisher’s exact test, Shapiro–Wilk test, Mann–Whitney (*U*) test, and Kruskal–Wallis (*H*) test, and binary logistic regression test were all carried out to evaluate the association between mucormycosis incidence and outcomes and its demographic and medical risk factors. All inferential tests were performed with the assumptions of confidence interval (*CI*) 95% and significance level (*Sig.*) < 0.05.

## 3. Results

### 3.1. Case Series

The reported patients in this case series were diagnosed with mucormycosis concurrently or immediately after SARS-CoV-2 infection, which was confirmed by the reverse transcription-polymerase chain reaction (RT-PCR) test. The patients with inadequate clinical or radiographic findings were excluded. None of the reported cases was vaccinated against SARS-CoV-2, and all of them were symptomatic when infected with COVID-19. The reported patients presented various levels of oxygen saturation (SpO2) ranging from 80% to 90%. The summary of the included patients’ characteristics and outcomes is presented in Table 1.

#### 3.1.1. Case No. 1

A 56-year-old male patient presented with a painful lesion in the hard palate for four days. Additionally, he had a fever and neurological symptoms. The patient had diabetes and had recently recovered from COVID-19.

The clinical examination shows a 2.5*1.5 cm sharp limited, necrotic, and ulcerative lesion in the left side of the hard palate. Radiological investigation using cone-beam computed tomography (CBCT) indicated hypertrophied nasal conchae with sinus involvement and bony erosions in the palate and alveolar region cortical bone.

**Figure 1:**
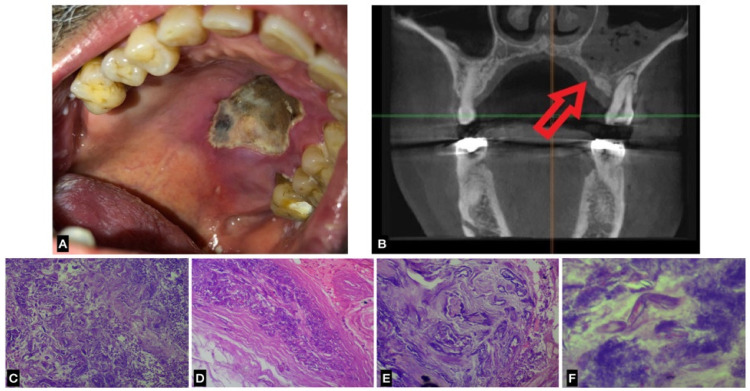
Case 1 with mucormycosis: (**A**) intra-oral photo shows the ulcerative and necrotic lesion in the left hard palate; (**B**) Cone-beam computed tomography (CBCT) in a coronal view showing opacification of the maxillary sinus, and irregular destruction of the palate (red arrow) and alveolar cortices on the left side. (**C**–**F**) Histological findings show a microphotograph of a ribbon-like organism with hyphae bent at a right-angle using H and E staining.

The histological findings of a biopsy specimen from the lesion confirmed the diagnosis of mucormycosis. The patient was hospitalised and improved following antimycotic medication using LAmB with a dosage of 4 mg/kg/day. This case is shown in Figure 1.

#### 3.1.2. Case No. 2

A diabetic 61-year-old female patient presented with a history of painful facial swelling around the right eye for two days. Additionally, she was febrile with neurological symptoms. Two weeks earlier, the patient had recovered from COVID-19.

**Figure 2:**
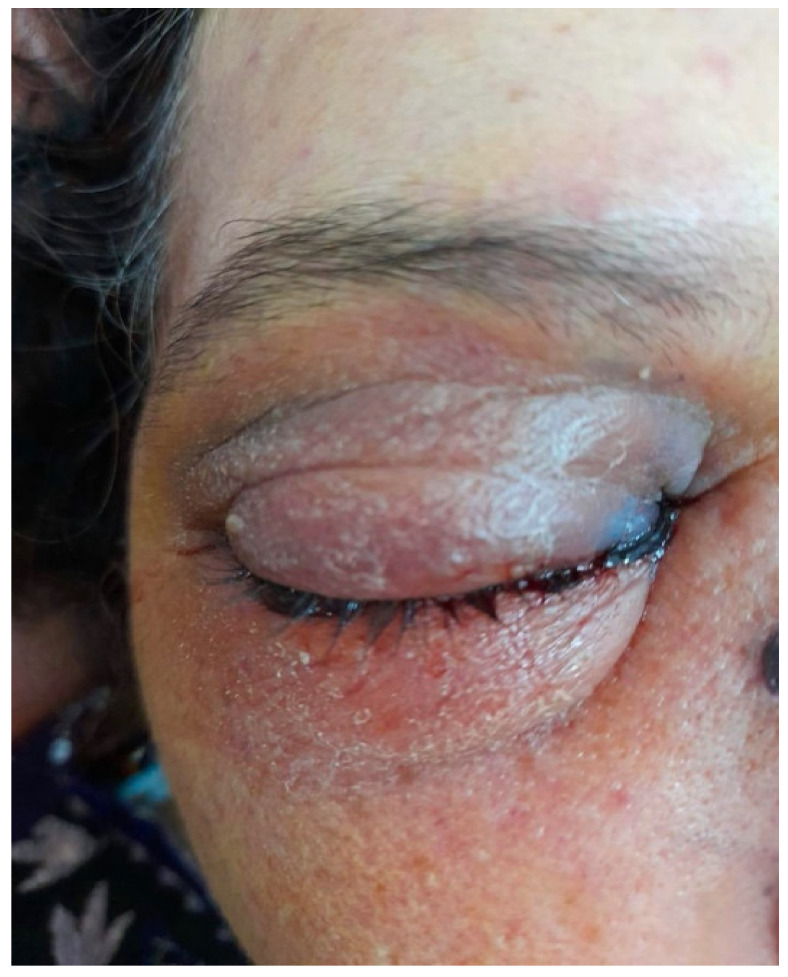
Extra-oral image of the Case No. 2 revealed a swelling around the right orbit.

The clinical examination shows erythema and facial swelling around the right eye. The eye was closed entirely with extensive eye movement restrictions. This case is shown in Figure 2.

The radiographic investigation using computed tomography (CT) showed hypertrophied nasal conchae with sinus involvement and facial bone erosions. The clinical, radiographic, and histological findings indicated the suspected diagnosis of “mucormycosis”.

Therefore, the patient was hospitalised and received a preemptive treatment with antimycotic medication using LAmB with a dosage of 4 mg/kg/day. The necrotic bone was surgically removed under general anaesthesia, followed by laser sterilisation by diode laser. Further laser biostimulation was used to improve soft tissue healing. The patient improved during the subsequent post-operative follow-up.

#### 3.1.3. Case No.3

A diabetic and hypertensive 66-year-old male patient who had recovered from COVID-19 four weeks before presented with pain, nasal stiffness, and a foul smell that developed one week ago. The patient was initially presented to an ENT specialist who treated the case as chronic sinusitis without positive progress.

The clinical examination showed erythema, induration, black discolouration over the side of the nose, and an ulcer on the lower left eyelid. The eye was closed with movement restrictions. The radiological investigations using a facial CT showed bone destruction in the related region.

**Figure 3:**
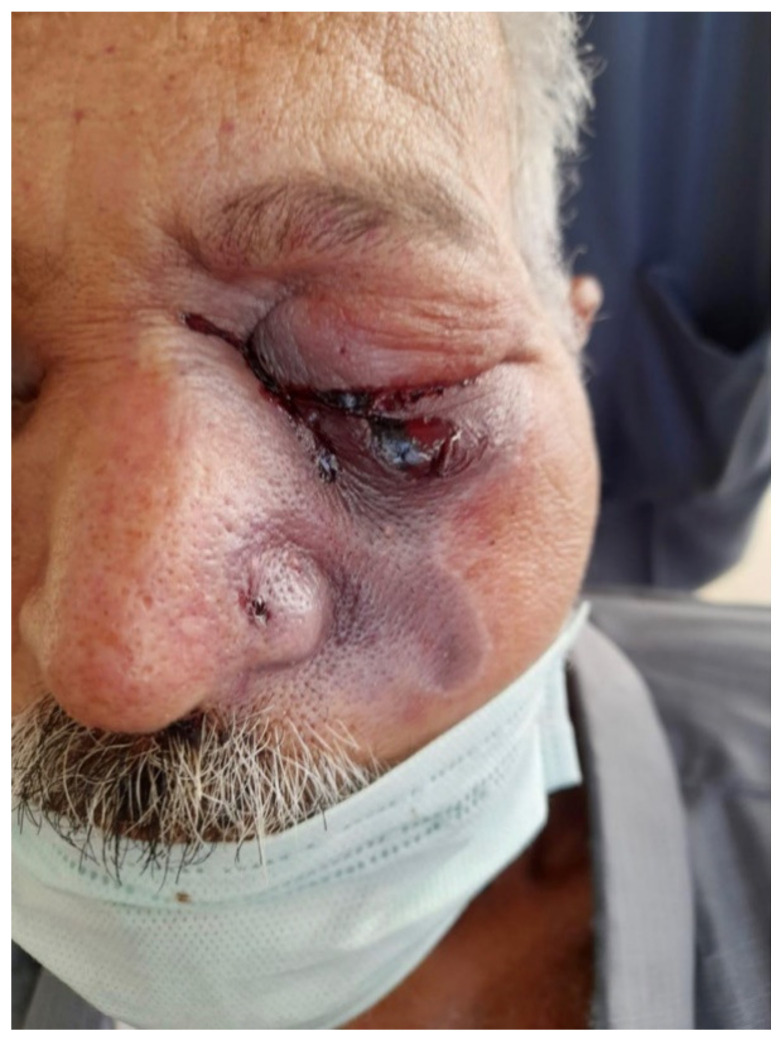
Extra-oral image of Case No. 3 revealed erythema, black discolouration, and an ulcer on the lower eyelid of the left eye.

Under hospitalisation and general anaesthesia, the necrotic bone was surgically removed, and a soft tissue laser was used to improve healing. The patient improved following antimycotic treatment using LAmB with a dosage of 4 mg/kg/day. This case can be seen in Figure 3.

#### 3.1.4. Case No.4

A diabetic and cardiac 52-year-old male patient who had recovered from COVID-19 four weeks before presented with pain and ulceration on the side of the nose that developed ten days ago. The initial diagnosis presented by a dermatologist was skin abrasion, but the therapy was without positive progress.

The clinical examination showed erythema, swelling, induration, and a necrotic patch with ulceration and extensive destruction of the right external nasal wall. The radiographic examination using facial CT reported hypertrophied nasal conchae, sinus involvement, and bone destruction to both the maxillary sinus and medial orbital wall.

The patient was immediately hospitalised and started the same surgical and medical treatment procedures as the last case. The follow-up examination exhibited stable and positive progress.

#### 3.1.5. Case No.5

A 58-year-old male patient with a positive SARS-CoV-2 PCR test presented febrile and cellulitis around the left eye for two weeks. The clinical examination showed eye movement restrictions. The radiological examination using CT imaging showed sinusoidal sinus abscess invaded orbital bone and eye with compression on the optic nerve. A PCR test was positive for Mucorales. Under ICU hospitalisation and general anaesthesia, surgical removal of the necrotic bone was performed. The patient showed stable progress following antimycotic treatment using LAmB with a dosage of 4 mg/kg/day.

#### 3.1.6. Case No.6

A 68-year-old female patient was presented to the ICU with acute ischemic stroke. A SARS-CoV-2 PCR test was positive. The patient’s anamnesis included diabetes mellitus and stable angina. The radiographic investigation using CT revealed invasive fungal sinusitis. The PCR test was positive for Mucorales. Therefore, the patient received antimycotic treatment using LAmB with a dosage of 4 mg/kg/day, and she improved subsequently.

#### 3.1.7. Case No.7

A 47-year-old male presented with dyspnea and generalised fatigue. The patient had no past medical history except mild COVID-19 18 days before. While the SARS-CoV-2 PCR test was negative, the chest CT showed typical ground-glass opacity (GGO) due to the viral infection.

According to the dyspnea, the patient was admitted to ICU. Suddenly, he developed a disturbed conscious level, disorientation, and later on, fixed pupil unilaterally. Intra-orally, the patient showed severe oral fungus, and the eye showed black colouration. The clinical, radiographic, and Mucorales positive PCR results confirmed the diagnosis of mucormycosis. Consequently, the patient improved following antimycotic treatment using LAmB with a dosage of 4 mg/kg/day.

### 3.2. Literature Review

#### 3.2.1. Demographic Characteristics

A total of 191 CAM patients were reported by 43 studies until 30 May 2021 [46,47,48,49,50,51,52,53,54,55,56,57,58,59,60,61,62,63,64,65,66,67,68,69,70,71,72,73,74,75,76,77,78,79,80,81,82,83,84,85,86,87,88]. Case reports were the most prevalent study design (70.7%), followed by case series (26.8%) and cross-sectional studies (2.4%). The majority of patients were males (74.4%), and their mean age was 55.08 ± 14.78 (14–88) years old.

**Figure 4:**
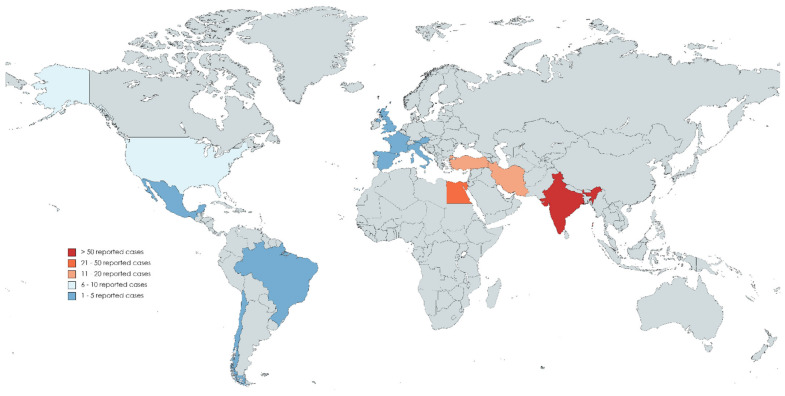
Global Distribution of COVID-19-associated Mucormycosis (CAM) Reported Cases, January 2020–May 2021.

On analyzing the sources of the reported cases, India was the most contributing country (55.5%) [46,47,48,49,50,58,69,77,80,81,82,84,85,86,87,88], followed by Egypt (17.8%) [72,74], Iran (9.9%) [63,64,70,78], Turkey (6.3%) [65,71], and the United States of America (4.7%) [52,53,54,55,56,57,59,75,76]. See Figure 4.

**Figure 5:**
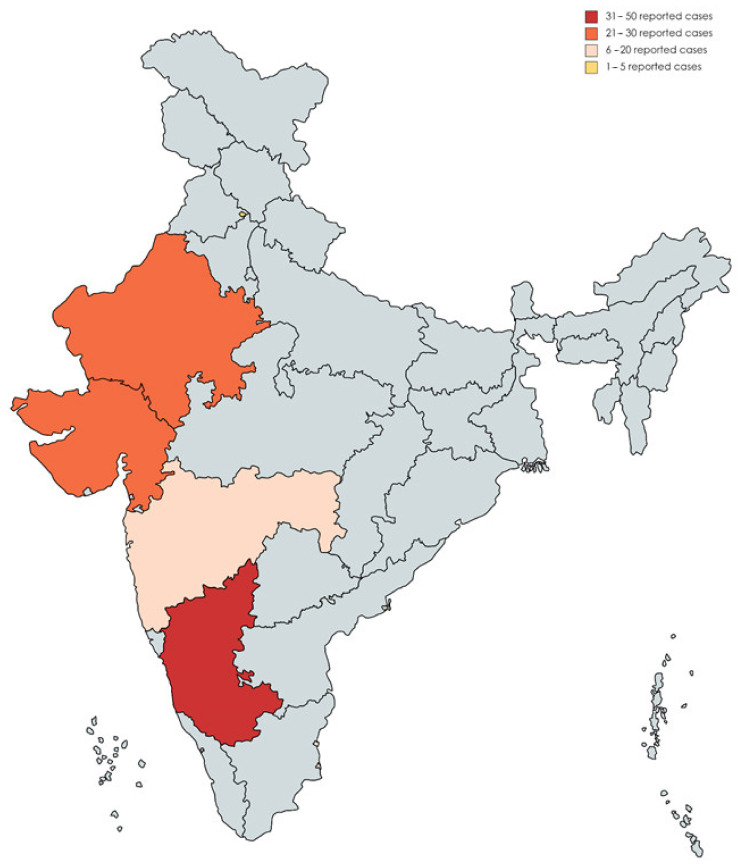
Geographic Distribution of COVID-19-associated Mucormycosis (CAM) Reported Cases in the Republic of India, January 2020–May 2021.

Across the Republic of India, the most contributing region was Karnataka (39.6%), followed by Rajasthan (26.4%), Gujarat (19.8%), Maharashtra (7.5%), Puducherry (5.7%), and Chandigarh (0.9%) Figure 5.

According to the recent classification of the World Bank for the fiscal year 2021, the vast majority of cases were from lower-middle-income economies (83.2%), followed by high-income economies (8.9%) and upper-middle-income economies (7.9%). There were no cases reported from low-income economies [89]. See Table 2.

#### 3.2.2. Anamnestic Characteristics

Out of the 191 reported cases in the literature, 110 cases were described in detail; therefore, their data were used in the downstream analyses (Supplementary File S1).

The vast majority of CAM patients (90.9%) had at least one medical comorbidity, including diabetes mellitus (79.1%), chronic hypertension (30%), renal disease/failure (13.6%), cardiovascular disease (10%), thyroid disease (5.5%), and organ transplantation (2.7%). See Table 3.

The severity of the COVID-19 infection was classified according to the Australian guidelines for the clinical care of people with COVID-19 [90]. The clinical symptoms and severity were reported in only 44.5% of the patients. Of those patients with available information, 40.8% were severe, 26.5% moderate, 18.4% mild, and 14.3% critic cases.

The most common medications for COVID-19 management were steroids (64.5%), followed by Remdesivir (18.2%), antibiotics (12.7%), and Tocilizumab (5.5%). Hydroxychloroquine (1.8%) was rarely used.

#### 3.2.3. Clinical Characteristics

The onset of mucormycosis varied remarkably across the reported cases, with 44 cases (40%) being diagnosed with mucormycosis and COVID-19 concurrently. In the rest of the cases (60%), the onset of CAM diagnosis ranged between 1 and 42 days with a mean value of 13.43 ± 8.87 days.

On reviewing the clinical features of the reported cases, most of them were affected unilaterally (90%). In the maxillofacial region, orbits (74.8%) were the most affected location, followed by paranasal sinuses (72%), the nasal cavity (18.5%), and the palate (13%). Additionally, the central nervous system (20.4%), lungs (9.3%), heart (0.9%), gastrointestinal tract (0.9%), and kidney (0.9%) were also affected to various degrees.

The ophthalmologic signs and symptoms included loss of/decrease in vision (44.4%), ptosis (41.7%), ophthalmoplegia (17.6%), fixed/decreased pupil/ocular movement (12%), chemosis (10.2%), and diplopia (0.9%). The otolaryngologic signs and symptoms included sinusitis (15.7%) and rhinorrhea (3.7%). The orofacial signs and symptoms included facial edema (29.6%), palatal eschar (13%), numbness (1.9%), and facial cellulitis (0.9%). See Table 4.

#### 3.2.4. Paraclinical Characteristics

Specimen biopsies were used to confirm mucormycosis infection in 93.6% of the reported cases. The radiographic aids, including cone-beam computed tomography systems (CBCT), computed tomography (CT), and magnetic resonance imaging (MRI), were used to validate the diagnosis in 90.9% of the cases.

The histopathologic assessment was used in only seven cases, and autopsies were the source of information for three cases. The microbiological examination revealed that *Mucor* was the most isolated genus (54.2%), followed by *Rhizopus* (39%) and *Lichtheimia* (3.4%). See Table 5.

#### 3.2.5. Management and Outcome

Surgical debridement was used to treat 77.9% of the reported cases, while medical treatments (antifungals) were used in 93.5% of the cases. The most commonly administered medication was amphotericin B (92.5%), followed by posaconazole (4.7%) and isavuconazole (3.7%).

While the outcome of the two cases was not reported, 48.1% of the reported cases improved, 46.3% died, 5.6% remained unchanged Table 6.

#### 3.2.6. Risk of Mortality

Despite the majority of CAM patients were males (74.4%), the incidence of mortality was not significantly different (*Sig.* = 0.640) between male patients (47.6%) and female patients (42.3%). The mortality level among patients aged 55 years old or below (51.8%) and the patients from upper-middle- and high-income economies (75%) was higher (*Sig.* = 0.272 and <0.001) than the patients aged above 55 years old (41.2%) and the patients from low- and lower-middle-income economies (34.2%).

The CAM patients with medical comorbidities did not have a significant difference (*Sig.* = 1.000; 2-S Fisher’s exact test) in terms of mortality (90%) and survival (91.4%) rates. Diabetic patients had a significantly (*Sig.* = 0.012) lower mortality rate (68%) than survival rate (87.9%). The CAM patients who received steroids to treat their COVID-19 infection had similar survival (65.5%) and mortality (64%) rates.

The concurrent diagnosis of COVID-19 and mucormycosis was associated with a mortality rate of 37.2%, while the latent diagnosis of mucormycosis was associated with a mortality rate of 50.9%. The CAM patients with bilateral manifestations (66.7%) had a higher mortality rate compared to the patients with unilateral manifestations (44.3%). Orbits, paranasal sinuses, and the central nervous system were not associated with higher mortality rates. The patients with lung manifestations had a significantly (*Sig.* = 0.041; 2-S Fisher’s exact test) higher rate of mortality (16.7%) than survival (3.4%). The good news is that the patients with nasal cavity manifestations had a significantly (*Sig.* = 0.012) higher rate of survival (27.6%) than mortality (8.3%).

Although there was a trend toward survival among the patients who were diagnosed using specimen biopsies, there was no significant difference (*Sig.* = 0.177) between their survival (98.3%) and mortality (91.8%) rates. Similarly, using histopathologic assessment had no significant difference (*Sig.* = 0.122) between survival (10.3%) and mortality (2%) rates. *Rhizopus*-infected cases had no significant difference (*Sig.* = 0.562) between their survival (35.5%) and mortality (42.9%) rates. Likewise, *Mucor*-infected cases had no significant difference (*Sig.* = 0.535) between their survival (58.1%) and mortality (50%) rates.

Surgical debridement had a trend towards survival; however, the difference between survival (55.7%) and mortality (43.5%) rates was not statistically significant (*Sig.* = 0.302). All the administered antifungals were associated with slightly higher levels of survival than mortality. See Table 7.

After running binary logistic regression for the binary outcome of mortality, the patients from upper-middle- and high-income economies had an increased odds ratio of mortality, 5.769 (CI 95%: 2.276–14.622). See Table 8.

Diabetes was not associated with an increased odds ratio of morality, 0.292 (CI 95%: 0.109–0.784). While the lung was associated with an increased odds ratio of mortality 5.600 (CI 95%: 1.129–27.785), the nasal cavity was associated with a decreased odds ratio of mortality 0.239 (CI 95%: 0.074–0.772).

For a better understanding of the increased mortality likelihood among the patients from upper-middle and high-income economies, adjustment for COVID-19 clinical severity yielded significantly increased mortality odds for those from upper-middle and high-income economies 8.098 (CI 95%: 1.652–39.685; *Sig.* = 0.010).

## 4. Discussion

The CAM incidence was higher among males than females, and this difference was consistently found in our case series (71.4%) and the reviewed literature (74.4%). Moreover, this finding is comparable to the previously published epidemiologic studies during the pre-COVID-19 era [91]. It remains unclear why males have a higher frequency of mucormycosis; however, observations of the protective function of estrogen in the development of paracoccidioidomycosis may explain this male susceptibility for mycosis. The possible function of estrogen in the transmission of mucormycosis infection has not yet been investigated [91,92].

India (55.5%), Egypt (17.8%), Iran (9.9%), and Turkey (6.3%) are currently the countries with the most reported CAM cases in the literature. A few months before the COVID-19 outbreak, a review article reported an increase in the prevalence of mucormycosis among Asian countries [93]. In India, mucormycosis patients increased from 24.7 cases per year (1990–2007) to 89 cases per year (2013–2015) in a single tertiary-care hospital [94]. In Iran, the prevalence increased from 9.7% in 2008 to 23.7% in 2014 [95]. Mucormycosis has been more prevalent in the Middle East and North Africa region during recent decades, coinciding with an increase in the number of individuals suffering from underlying medical problems connected with this illness [96]. However, the actual number of mucormycosis cases can also be underestimated, especially in developing countries. An explanation for the increasing mucormycosis incidence in the countries mentioned above may be related to the climate. Previous studies documented a high prevalence of mucormycosis in the tropical and subtropical climate zones, especially in autumn [17,97].

The background prevalence of mucormycosis in India had been estimated to be 80 times the average prevalence globally [98,99]. However, there is a lack of evidence on the background prevalence of mucormycosis in Egypt; it had been suggested to be above the global average due to the fragility of the health system and the heavy national burden of diabetes mellitus and other immunosuppressive conditions that resemble the situation in India [100]. Both India and Egypt were strongly hit by the second and third waves of the COVID-19 pandemic in late 2020 and the first half of 2021, and this sharp increase may have affected people’s access to health services and would have increased the probability of diabetic patients or immunocompromised cohorts to get sick by COVID-19 [101].

The majority of CAM patients in the current review (90.9%) had at least one medical comorbidity, including diabetes mellitus (79.1%), chronic hypertension (30%), renal disease/failure (13.6%), cardiovascular disease (10%), thyroid disease (5.5%), and solid organ transplantation (2.7%). Compared to the medical risk factors described by Roden et al. in 2005, who found that mucormycosis was common among patients with diabetes mellitus (36%), malignancies (17%), and solid organ transplantation (7%), the outcomes of this literature review revealed that the medical risk factors of CAM include additional comorbidities that might be related to COVID-19 primarily [91]. In addition, 64.5% of the patients had a history of consuming steroids. These findings are similar to the data from the case series presented in this study and most of the published cohort studies and meta-analyses. As phagocytes typically kill Mucorales through the production of oxidative metabolites, it has been demonstrated in clinical studies that these phagocytes are the most crucial host defensive mechanism against mucormycosis [102]. Therefore, patients with medical comorbidities that affect phagocytes’ number and function are at increased risk of sustaining mucormycosis. These include neutropenic patients, hyperglycemia and acidosis, corticosteroid treatment and recently COVID-19 infections. However, the specific processes by which ketoacidosis, diabetes, or steroids affect the function of these phagocytes are yet unclear [103].

### 4.1. Is CAM an Interaction between SARS-CoV-2 Virus and Mucor Fungus?

Fungal infections in COVID-19 patients have been reported in several studies since the pandemic outbreak. Oropharyngeal candidiasis was reported in 12.9% of the critically ill COVID-19 patients with oral complications, and they were even reported in non-severe cases [4,5]. SARS-CoV-2-associated pulmonary aspergillosis (CAPA) was firstly reported and was mainly associated with acute respiratory distress syndrome (ARDS) [8]. Later, CAM cases were reported in different populations and patients with all COVID-19 severity grades. The pathogenesis of both fungal infections is still unclear; however, virus/fungus interaction mechanisms may contribute to the development of disease patterns [104]. The invasion of the SARS-CoV-2 virus may lead to danger-associated molecular patterns (DAMPs), which are known to play a vital role in the pathogenesis of fungal diseases [105]. However, this study also found CAM in mild COVID-19 cases, thus challenging this theory [105].

### 4.2. Are the Medical Anamneses and Treatment Protocols the Missing Keys?

Another possible explanation of the correlation between COVID-19 and mucormycosis may be the treatment modalities for severe COVID-19. CAM can be a side effect or even a superinfection due to corticosteroids, anti-IL-6 therapy, and antibiotics to manage severely ill COVID-19 patients. This review revealed that 64.5% of the patients took steroids, 12.7% antibiotics, and 5.5% Tocilizumab. However, 24.58% of the patients included in this review did not take any previously mentioned medications.

Diabetes, which is strongly associated with mucormycosis infection and a significantly increased risk of SARS-CoV-2 infection, may have disastrous consequences for the surrounding community [106]. The current study can prove this finding as 68% of the mortality and 87.9% of the survivals were diabetic patients [106].

### 4.3. Is CAM a Healthcare-Associated Condition?

Several studies reported that mucormycosis was a result of hospital-acquired disease [107,108]. Contamination of umbilical catheter, dressing materials, wooden tongue depressors, and bandages have been documented as a source of fast spreading of the disease among the hospitalised patients [108,109,110]. These documented accidents from the past highlighted a potential reason for the present crisis. Hospitals that were overburdened with COVID-19 patients and the lack of enough ventilators may encourage the spread of the disease [106].

### 4.4. Strengths

This study is the first to analyse the risk factors of CAM mortality, which were not identical to the risk factors of CAM incidence. While diabetes, organ transplantation, and steroids were the most common risk factors among CAM patients, the risk of mortality was not significantly higher among the patients who received steroids and those who had diabetes and organ transplantation. These results should be interpreted cautiously because they are based on observational evidence prone to reporting bias.

Our case series supported the male predominance of CAM incidence. To the best of the authors’ knowledge, this study is the first to reveal that the risk of CAM mortality is higher in upper-middle- and high-income countries, which may reflect less training capacity of medical staff in the developed economies to manage fungal infections due to their rarity or can simply be a result of reporting bias. The academic physicians in low- and lower-middle-income countries might have been more inclined to report the survived cases rather than display the effectiveness of their treatment protocols to treat the CAM cases.

### 4.5. Limitations

The first limitation of this case series is the time and location constraints where the cases were reported; therefore, they may not necessarily reflect the entire CAM population on a national or global level. Additionally, a part of this study is naturally limited, as a literature review, by the breadth and robustness of the included primary studies, which were mainly case reports and case series; therefore, the conclusions regarding the risk factors of CAM mortality should be interpreted carefully as the evidence coming from descriptive observational studies usually retain a low level of certainty.

### 4.6. Implications

The findings of this study confirm that the use of steroids was not associated with an increased risk of CAM mortality; therefore, the use of steroids in managing severe and critical COVID-19 patients should not be discontinued.

The increased risk of mortality in the cases with lung involvement and *Rhizopus* species suggests that proactive screening for critically ill patients is necessary. The decreased risk of mortality among the patients who were managed surgically and those who received amphotericin B and posaconazole confirm the effectiveness of these treatment modalities.

## 5. Conclusions

Heretofore, 191 CAM cases were reported worldwide, of which 74.4% were males, 83.2% were from low-middle income countries, and 51.4% were aged 55 years old or below. Diabetes mellitus (79.1%), chronic hypertension (30%), and renal disease/failure (13.6%) were the most common medical comorbidities, while steroids (64.5%) were the most frequently prescribed medication for COVID-19, followed by Remedsivir (18.2%), antibiotics (12.7%), and Tocilizumab (5.5%).

Diabetes, steroids, and Remedsivir were not associated with an increased risk of mortality, thus confirming that the use of steroids to manage severe and critical COVID-19 patients should not be discontinued.

Lung involvement, bilateral manifestation, and *Rhizopus* spp. isolation were associated with increased mortality risk, thus confirming that proactive screening should be followed for critically ill patients. Finally, surgical management and antimycotic medications, e.g., amphotericin B and posaconazole, were associated with a decreased mortality risk, thus suggesting their effectiveness.

## Figures and Tables

**Table 1:** Anamnestic, Clinical, and Paraclinical Features of COVID-19-associated Mucormycosis Cases in Egypt.

	Case No. 1	Case No. 2	Case No. 3	Case No. 4	Case No. 5	Case No. 6	Case No. 7
Age, Gender	56, Male	61, Female	66, Male	52, Male	58, Male	68, Female	47, Male
Anamnesis	Diabetes Mellitus	Diabetes Mellitus	Diabetes mellitus; chronic hypertension	Diabetes mellitus; cardiovascular disease	Diabetes mellitus	Diabetes mellitus; Stable angina	Healthy
Blood Glucose ^(I)^	350 mg/dL	400 mg/dL	320 mg/dL	300 mg/dL	450 mg/dL	300 mg/dL	160 mg/dL
Vaccinated	No	No	No	No	No	No	No
COVID-19 Symptoms	Fever; cough; shortness of breath, diarrhea	Fever; cough; SpO2: 85%	Fever; cough; diarrhea; SpO2: 80%	Fever; cough; shortness of breath; SpO2: 86%	Fever; shortness of breath; SpO2: 90%	Fever; cough; shortness of breath, diarrhea	Fever; cough; shortness of breath, diarrhea
COVID-19 Treatment	Azithromycin; dexamethasone; salbutamol sulphate (Farcolin); paracetamol; acetaminophen (Amol); enoxaparin sodium (Clexane); zinc	Azithromycin; dexamethasone; salbutamol sulphate (Farcolin); paracetamol; acetaminophen (Amol); enoxaparin sodium (Clexane); zinc	Azithromycin; dexamethasone; salbutamol sulphate (Farcolin); paracetamol; acetaminophen (Amol); enoxaparin sodium (Clexane); zinc	Azithromycin; dexamethasone; salbutamol sulphate (Farcolin); paracetamol; acetaminophen (Amol); enoxaparin sodium (Clexane); zinc	Azithromycin; dexamethasone; salbutamol sulphate (Farcolin); paracetamol; acetaminophen (Amol); enoxaparin sodium (Clexane); zinc	Azithromycin; dexamethasone; salbutamol sulphate (Farcolin); paracetamol; acetaminophen (Amol); enoxaparin sodium (Clexane); zinc	Azithromycin; dexamethasone; salbutamol sulphate (Farcolin); paracetamol; acetaminophen (Amol); enoxaparin sodium (Clexane); zinc
Onset ^(II)^	After 4 days of COVID-19 recovery	After two weeks of COVID-19 recovery	After four weeks of COVID-19 recovery	After four weeks of COVID-19 recovery	After two weeks of COVID-19 diagnosis	After three days of COVID-19 diagnosis	After 18 days of COVID-19 diagnosis
Clinical Features	Fever; necrosis and ulceration of hard palate (Figure 1)	Fever; unilateral periorbital swelling (Figure 2)	Nasal stiffness; oral malodor; periorbital ulceration; nasal sidewalls discolouration	Nasal sidewalls ulceration and necrosis	Fever; periorbital cellulitis	GCS deterioration	Dyspnea, GCS deterioration; oral fungus; periorbital discoloration
Neurologic Symptoms	Slurred speech; perioral numbness	Slurred speech; seizure; periorbital numbness	Numbness in the affected side of the face; lethargy	Slurred speech; periorbital numbness	No neurologic symptoms were reported	Seizure; coma	Seizure; coma
Mucormycosis	Rhino-cerebral	Rhino-cerebral	Rhino-cerebral	Rhino-cerebral	Rhino-cerebral	Rhino-cerebral	Rhino-cerebral
Radiographic Features	**CBCT**: Hypertrophied nasal conchae; opacified maxillary sinus; and irregular palatal destruction (Figure 2)	**CT**: Hypertrophied nasal conchae; sinus involvement with opacification; and bony erosions	**CT**: Facial bone destruction (Figure 3); sequestration with empty osteocytic lacunae	**CT**: Hypertrophied nasal conchae; sinus involvement and bilateral destruction of the maxillary sinus	**CT**: Sinusoidal sinus abscess invaded the orbital bone compressing the optic nerve (Figure 1)	**CT**: invasive sinusitis with opacities and bony erosion in the anterior wall;	**CBCT**: CT showed hypertrophied nasal conchae, sinus involved with opacities and bony erosion in the anterior wall;
Microbiologic Features	**Palatal Specimen Biopsy**: ribbon-like spores; branching and non-septate bends; angiodestruction (Figure 3)	**Biopsy Specimen**: ribbon-like spores; branching and non-septate bends; angiodestruction, areas of bony sequestration with empty osteocytic lacunae.	**Biopsy Specimen**: ribbon-like spores; branching and non-septate bends; angiodestruction, areas of bony	**Biopsy specimen**: ribbon-like spores; branching and granulation tissue infiltrated with inflammatory	*N/A*	**RT-PCR**: positive for Mucorales	**RT-PCR**: positive for Mucorales
Hospitalised	Yes	Yes	Yes	Yes	Yes	Yes	Yes
Treatment	Hospitalisation; LAmB (4 mg/kg/day); and surgical debridement	Hospitalisation; LAmB (4 mg/kg/day); and surgical debridement	Hospitalisation; LAmB (4 mg/kg/day); surgical debridement; and soft tissue laser to improve healing	Hospitalisation; LAmB (4 mg/kg/day); surgical debridement; and soft tissue laser to improve healing	Hospitalisation; LAmB (4 mg/kg/day); and surgical debridement	Hospitalisation; LAmB (4 mg/kg/day)	Hospitalisation; LAmB (4 mg/kg/day)
Follow-up	4 weeks	6 weeks	5 weeks	4 weeks	5 weeks	4 weeks	4 weeks
Outcome	Improved	Improved	Improved	Stable	Stable	Improved	Improved

SpO2: oxygen saturation level; GCS: Glasgow Coma Scale; CBCT: cone-beam computed tomography; CT: computed tomography; RT-PCR: reverse transcription-polymerase chain reaction. ^(I)^ The blood glucose level was estimated on the day of admission. ^(II)^ The recovery from COVID-19 is defined as the patient obtaining two successive negative RT-PCR results, so the recovery date represents the second negative RT-PCR result.

**Table 2:** Demographic Characteristics of COVID-19-associated Mucormycosis (CAM) Cases, Global Evidence Synthesis, January 2020–May 2021.

Variable	Outcome	Reported Frequency (*n*)	Valid Percentage (%)
Study Design	Case Report	29	70.7
	Case Series	11	26.8
	Cross-sectional	1	2.4
Gender	Female	34	25.6
	Male	99	74.4
Age	≤55 years-old	56	51.4
	>55 years-old	53	48.6
Country	Austria	1	0.5
	Brazil	1	0.5
	Chile	1	0.5
	Egypt	34	17.8
	France	1	0.5
	India	106	55.5
	Iran	19	9.9
	Italy	1	0.5
	Mexico	2	1
	Spain	2	1
	Turkey	12	6.3
	UK	2	1
	USA	9	4.7
Economic Rank	Low income	0	0
	Lower-middle income	159	83.2
	Upper-middle income	15	7.9
	High income	17	8.9

**Table 3:** Anamnestic Characteristics of COVID-19-associated Mucormycosis (CAM) Cases, Global Evidence Synthesis, January 2020–May 2021.

Variable	Outcome	Reported Frequency (*n*)	Valid Percentage (%)
Comorbidities	Diabetes Mellitus	87	79.1
	Chronic Hypertension	33	30
	Renal Disease/Failure	15	13.6
	Cardiovascular Disease	11	10
	Thyroid Disease	6	5.5
	Asthma	4	3.6
	Hematologic Malignancy	4	3.6
	Organ Transplantation	3	2.7
	Anxiety Disorder	2	1.8
	Hepatic Disease	2	1.8
	Cerebral Infraction	1	0.9
	Chronic Obstructive Pulmonary Disease	1	0.9
	Hyperlipidemia	1	0.9
	Myelodysplastic Syndrome	1	0.9
	Tuberculosis	1	0.9
	Total	100	90.9
COVID-19 Severity	Mild	9	18.4
	Moderate	13	26.5
	Severe	20	40.8
	Critic	7	14.3
	Not Reported	61	55.5
COVID-19 Medication	Steroids	71	64.5
	Remdesivir	20	18.2
	Antibiotics	14	12.7
	Tocilizumab	6	5.5
	Oseltamivir	3	2.7
	Lopinavir/Ritonavir	2	1.8
	Hydroxychloroquine	2	1.8

**Table 4:** Clinical Characteristics of COVID-19-associated Mucormycosis (CAM) Cases, Global Evidence Synthesis, January 2020–May 2021.

Variable	Outcome	Reported Frequency (*n*)	Valid Percentage (%)
Side	Unilateral	81	90
	Bilateral	9	10
Location	Orbits	80	74.8
	Paranasal Sinuses	77	72
	Central Nervous System	22	20.4
	Nasal Cavity	20	18.5
	Lung	10	9.3
	Palate	2	1.9
	Heart	1	0.9
	Gastrointestinal Tract	1	0.9
	Kidney	1	0.9
	Limb	1	0.9
Signs and Symptoms	Loss/Decrease of Vision	48	44.4
	Ptosis	45	41.7
	Facial Edema	32	29.6
	Necrotic Tissue	30	27.8
	Ophthalmoplegia	19	17.6
	Sinusitis	17	15.7
	Palatal Eschar	14	13
	Fixed/Decreased Pupil/Ocular Movement	13	12
	Chemosis	11	10.2
	Rhinorrhea	4	3.7
	Ulceration	3	2.8
	Pus	3	3.2
	Diplopia	1	0.9
	Facial Cellulitis	1	0.9

**Table 5:** Paraclinical Characteristics of COVID-19-associated Mucormycosis (CAM) Cases, Global Evidence Synthesis, January 2020–May 2021.

Variable	Outcome	Reported Frequency (*n*)	Valid Percentage (%)
Method	Biopsy	103	93.6
	CBCT/CT/MRI	100	90.9
	Histopathology	7	6.4
	Autopsy	3	2.7
Genus	*Mucor*	32	54.2
	*Rhizopus*	23	39
	*Aspergillus*	5	8.5
	*Lichtheimia*	2	3.4
	Not Reported	59	53.6

CBCT: cone-beam computed tomography; CT: computed tomography; MRI: magnetic resonance imaging.

**Table 6:** Management and Outcome of COVID-19-associated Mucormycosis (CAM) Cases, Global Evidence Synthesis, January 2020–May 2021.

Variable	Outcome	Reported Frequency (*n*)	Valid Percentage (%)
Surgical Intervention	Yes	81	77.9
	No	23	22.1
	Not Reported	6	5.5
Medication	Amphotericin B	99	92.5
	Posaconazole	5	4.7
	Isavuconazole	4	3.7
	Voriconazole	2	1.9
	Itraconazole	1	0.9
	Not Reported	3	2.7
Outcome	Improved	52	48.1
	Unchanged	6	5.6
	Death	50	46.3
	Not Reported	2	1.8

**Table 7:** Demographic, Anamnestic, Symptomatic, and Interventional Risk Factors of Mortality among COVID-19-associated Mucormycosis (CAM) Cases, Global Evidence Synthesis, January 2020–May 2021.

Variable	Outcome	Mortality (*n* = 50)	Survival (*n* = 58)	*Sig.*
Gender	Female	11 (22%)	15 (25.9%)	0.640
	Male	39 (78%)	43 (74.1%)	
Age	≤55 years-old	29 (58%)	28 (48.3%)	0.272
	>55 years-old	21 (42%)	30 (51.7%)	
Economy	Low and Lower-middle Income	26 (52%)	50 (86.2%)	**<0.001**
	Upper-middle and High Income	24 (48%)	8 (13.8%)	
Comorbidities	Diabetes	34 (68%)	51 (87.9%)	**0.012**
	Chronic Hypertension	16 (32%)	17 (29.3%)	0.762
	Renal Disease/Failure	8 (16%)	7 (12.1%)	0.556
	Cardiovascular Disease	5 (10%)	6 (10.3%)	0.953
	Thyroid Disease	3 (6%)	3 (5.2%)	1.000 *
	Asthma	3 (6%)	1 (1.7%)	0.334 *
	Hematologic Malignancy	3 (6%)	1 (1.7%)	0.334 *
	Organ Transplantation	1 (2%)	2 (3.4%)	1.000 *
	Total	45 (90%)	53 (91.4%)	1.000 *
COVID-19 Medication	Steroids	32 (64%)	38 (65.5%)	0.869
	Remdesivir	8 (16%)	12 (20.7%)	0.532
	Antibiotics	8 (16%)	6 (10.3%)	0.383
	Tocilizumab	4 (8%)	2 (3.4%)	0.412 *
	Total	37 (82.2%)	41 (87.2%)	0.503
Onset	Concurrent (0 days)	16 (37.2%)	27 (62.8%)	0.174
	Latent (≥ 1 day)	29 (50.9%)	28 (49.1%)	
Side	Unilateral	35 (44.3%)	44 (55.7%)	0.294 *
	Bilateral	6 (66.7%)	3 (33.3%)	
Location	Orbits	33 (68.8%)	45 (78.9%)	0.234
	Paranasal Sinuses	31 (64.6%)	44 (77.2%)	0.154
	Central Nervous System	11 (22.9%)	11 (19%)	0.618
	Nasal Cavity	4 (8.3%)	16 (27.6%)	**0.012**
	Lung	8 (16.7%)	2 (3.4%)	0.041 *
	Palate	0 (0%)	2 (3.4%)	0.500 *
Signs and Symptoms	Loss/Decrease of Vision	18 (37.5%)	29 (50%)	0.197
	Ptosis	22 (45.8%)	22 (37.9%)	0.411
	Facial Edema	13 (27%)	18 (31%)	0.656
	Necrotic Tissue	15 (31.3%)	15 (25.9%)	0.540
	Ophthalmoplegia	10 (20.8%)	9 (15.5%)	0.478
	Sinusitis	6 (12.5%)	11 (19%)	0.367
	Palatal Eschar	6 (12.5%)	8 (13.8%)	0.845
	Fixed/Decreased Pupil/Ocular Movement	6 (12.5%)	6 (10.3%)	0.727
	Chemosis	3 (6.3%)	7 (12.1%)	0.343 *
Diagnosis Method	Biopsy	45 (91.8%)	57 (98.3%)	0.177 *
	CBCT/CT/MRI	48 (98%)	50 (96.2%)	1.000 *
	Histopathology	1 (2%)	6 (10.3%)	0.122 *
Genus	*Mucor*	14 (50%)	18 (58.1%)	0.535
	*Rhizopus*	12 (42.9%)	11 (35.5%)	0.562
Surgical Intervention	Yes	35 (44.3%)	44 (55.7%)	0.302
	No	13 (56.5%)	10 (43.5%)	
Mucormycosis Medication	Amphotericin B	43 (89.6%)	55 (96.5%)	0.242 *
	Posaconazole	1 (2.1%)	4 (7%)	0.372 *
	Isavuconazole	1 (2.1%)	3 (5.3%)	0.623 *
	Voriconazole	1 (2.1%)	1 (1.8%)	1.000 *
	Itraconazole	0 (0%)	1 (1.7%)	1.000 *

Chi-squared test (*χ^2^*) and Fisher’s exact test (*) were used with a significance level (*Sig.*) of <0.05. The significant values are in bold font. CBCT: cone-beam computed tomography; CT: computed tomography; MRI: magnetic resonance imaging.

**Table 8:** Risk Factors of Mortality among COVID-19-associated Mucormycosis (CAM) Cases, Global Evidence Synthesis, January 2020–May 2021.

CAM Mortality Risk Factor (Predictor)		B (SE)	OR (CI 95%)	*Sig.*
Economy	Upper-middle and High Income	1.753 (0.474)	5.769 (2.276–14.622)	**<0.001**
Comorbidities	Diabetes	−1.232 (0.504)	0.292 (0.109–0.784)	**0.015**
Location	Nasal Cavity	−1.433 (0.599)	0.239 (0.074–0.772)	**0.017**
	Lung	1.723 (0.817)	5.600 (1.129–27.785)	**0.035**

The significant values are in bold font.

## Data Availability

The data that support the findings of this study are available from the corresponding author upon reasonable request.

## Supplementary Materials

The following are available online at https://www.mdpi.com/article/10.3390/jof7100837/s1, File S1: Extracted Data of CAM Cases Reported until May 2021.spv.
